# The Use of Cryogenic Size Reduction to Improve Purgeable Priority Pollutant Analyses in Soil Samples

**DOI:** 10.6028/jres.093.049

**Published:** 1988-06-01

**Authors:** J. H. Phillips, C. A. Potera, P. M. Michalko, J. H. Frost

**Affiliations:** Air Products and Chemicals, Inc., Allentown, PA 18195

Air Products and Chemicals has been investigating the use of cryogenics to improve the recoveries of priority pollutants from solid matrices. A significant number of benefits have been realized from cryogenic size reduction of materials prior to analysis. The precision and accuracy of trace organic analyses in solid matrices are improved by cryogenic techniques. Even the most difficult samples, from rocks to rubber, can be easily ground cryogenically. Cryogenic size reduction increases sample homogeneity, reduces analyte loss due to the heat of grinding, and improves extraction efficiency by increasing the surface area of the sample.

The Environmental Protection Agency (EPA) has proposed the Toxicity Characteristic Leaching Procedure (TCLP) to identify wastes which pose a hazard due to their potential to leach toxic species. Because the determination of volatile species was important, a Zero Headspace Extractor (ZHE) was designed to prevent volatile loss during leaching. A shortcoming of the proposed volatiles analysis is that a trade-off had been made between size reduction and volatile loss. The EPA recommends that only solids that are >9.5 mm or have a surface area of <3.1 cm^2^/g be reduced.

Clays were used as the synthetic soil matrix from which volatiles would be recovered. Clays have a number of unique properties which make them ideal candidates for adsorbing and retaining volatile organics. A hardened clay matrix was produced by combining one part kaolin clay, one part sepolite clay, one part cement, and two parts water. Twenty-one volatile organic priority pollutants were spiked into the clay matrix via the aqueous portion of the formulation. The spiking was performed at two concentrations, 20 mg/kg and 2.0 mg/kg in the solid matrix.

The spiked clay samples were reduced to 9.5 mm under ambient conditions, reduced to <200 mesh at ambient temperature, or reduced to <200 mesh at cryogenic temperature (−196 °C). After size reduction, the sample was extracted for 18 h in the ZHE according to TCLP protocol. The leachate was then analyzed by purge and trap GC/MS according to the EPA method #624.

At the 20 mg/kg spike concentration, recoveries ranged from 1.2% to 20%. Recoveries never approached 100% because a significant portion of volatiles was lost to the atmosphere during mixing and hardening of the clay-cement matrix. On a relative basis, cryogenic size reduction improved recoveries three fold over ambient and minimum size reduction techniques.

At the 2 mg/kg spike concentration, the effects of cryogenic size reduction were also quantified. Recoveries were improved an average of five times over ambient and minimum size reduction. Analysis precision, as measured by the percent relative standard deviation between three extractions, was also improved. The precisions of analyses were 5.9% for cryogenic size reduction (200 mesh @ −195 °C), 13% for minimum size reduction (9.5 mm @ 20 °C) and 25% for ambient size reduction (200 mesh @ 20 °C). These results indicated that when cryogenic size reduction was not possible, minimum size reduction was preferred.

Cryogenic grinding was clearly the best size reduction technique for the preparation of samples for volatile analysis. Advantages were increased analyte recovery, better sample homogeneity, and improved extraction efficiency.

